# Medical Journals: Role, Place, Issues, and Future Challenges

**DOI:** 10.3390/jcm13072059

**Published:** 2024-04-02

**Authors:** Emmanuel Andrès, Thierry Lavigne

**Affiliations:** 1 Department of Internal Medicine, University Hospital of Strasbourg, 67000 Strasbourg, France; 2 Département de Santé Publique, Hôpital Civil, University Hospital of Strasbourg, 67000 Strasbourg, France; thierry.lavigne@chru-strasbourg.fr

Society and humans are evolving at exponential speeds [1], with health and medicine following the same path. Digital technology and data are revolutionizing our world [2]. Information is becoming ephemeral, widely shared, incorrect, as well as fragmented, creating misinformation (fake news), with knowledge shifting and evolving along with uncertainty.

Against this backdrop, we can legitimately question the role and place of medical journals, like the *Journal of Clinical Medicine* (*J. Clin. Med.*), and their future. Younger-generation doctors and those still in training within the healthcare sector, in addition to wider society and our patients, are increasingly relying on data from popular science journals and websites, which often originate from non-professional sources (e.g., WhatsApp), other social networks (e.g., Facebook, X, or LinkedIn), or even artificial intelligence tools *(*e.g., CHATGPT).

With this in mind, we can seriously examine the role and place of medical journals and the issues and challenges that will impact them in the future.

## 1. Their Role and Place

**Figure 1:**
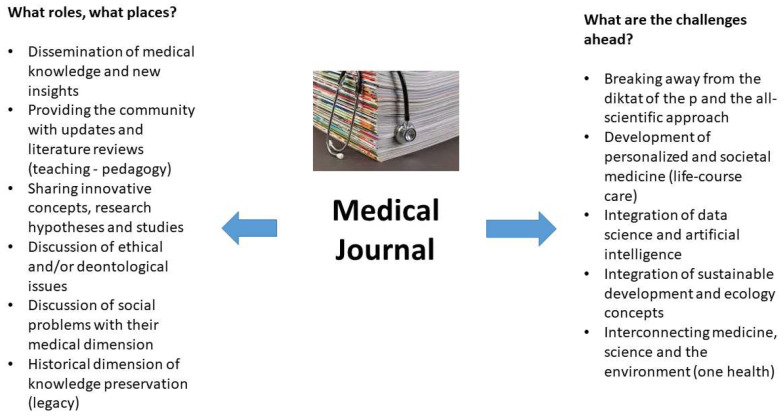
Roles, places, issues and future challenges for medical journals?

For decades, medical journals have contributed to the dissemination of new knowledge in various medical fields, notably through the publication of scientific articles reporting the results of original research (Figure 1) [3]. 

In this context, the *J. Clin. Med.* has established itself as one of the leading general medical journals alongside *The New England Journal of Medicine, The Lancet, The Annals of Internal Medicine, The British Medical Journal, The American Journal of Medicine, The Journal of Internal Medicine*, and the *European Journal of Medicine*. In the past year, *J. Clin. Med.* received an Impact Factor of 3.9 (Medicine, General & Internal, Q2 ranking 58/167), and a Cite Score of 5.4 (General Medicine, ranking 137/830).

Medical journals, such as those mentioned above, are indeed prominent platforms for the publication of scientific, translational, or clinical studies, as well as innovative research (Figure 1) [3]. These journals enable researchers to share their discoveries, explain their methodologies, and disseminate their results in a formal, peer-reviewed manner [4]. By publishing original articles, medical journals facilitate the exchange of innovative ideas and new concepts among researchers and practitioners.

Medical journals, such as the *J. Clin. Med.,* present articles on new clinical approaches and proofs of concepts, encouraging healthcare professionals to adopt innovative practices within their respective fields (Figure 1) [3]. All this fosters an intellectual environment conducive to innovation, built on robust, proven, and validated evidence.

The peer-review process, adopted by all the above-mentioned medical journals, guarantees the scientific quality of published articles, reinforcing the credibility of the information transmitted [4]. To date, all the above medical journals adhere to the recommendations of the International Committee of Medical Journal Editors (ICMJE). These recommendations are intended primarily for authors who are considering submitting their work for publication to an ICMJE member medical journal. These recommendations are designed to guarantee the quality of publications, compliance with ethical standards in research and other types of information (e.g., links to the pharmaceutical industry) published in medical journals [4]. The board of the *J. Clin. Med.* is particularly attentive to these recommendations.

Furthermore, medical journals provide regular updates or reviews on practice standards, clinical guidelines, and novel therapeutic approaches, thereby contributing to the dissemination of knowledge, in addition to the continuous improvement of healthcare and patient management (Figure 1) [3].

Given this context, medical journal articles are a valuable resource for continuing medical education, enabling healthcare professionals to engage with the latest advances in their field and develop their knowledge further (Figure 1) [4]. Indeed, medical journals, such as those listed above, are often categorized as teaching resources for the training of medical students, familiarizing them with current research and clinical practices. As such, they constitute an educational tool in the same sense as medical books.

Medical journals often address ethical and social issues related to new medical advances, encouraging reflection and discussion on the innovation’s ethical implications (Figure 1) [3,4]. Based on this scrutiny, medical journals offer essential guidance for practitioners and researchers alike, particularly with respect to thorny or controversial issues.

At this level, medical journals also provide historical documentation, recording the evolution of medical knowledge over time, thereby creating a legacy of scientific progress and societal positioning with respect to these issues (Figure 1) [3].

By bringing together articles from different medical disciplines, medical journals, such as the *J. Clin. Med*., foster interdisciplinary collaboration, which turns out to be paramount for innovative approaches to both research and healthcare practice.

Often available on the internet, in dematerialized form, medical journals offer global accessibility to medical knowledge, enabling healthcare professionals from different parts of the world to share and access crucial information, as well as to divulge their practices and experience with regard to the population issues addressed [3].

## 2. What Are the Challenges Ahead?

For decades, medical journals, such as those mentioned above, have been entirely subject to the strict standards of the “*p*”, inhibiting medicine from departing from the original art of medicine to fully become evidence-based medicine [5]. Given this context, it is crucial to emphasize that “*p* < 0.05” (statistical significance or the famous “statistically significant”) does not necessarily guarantee clinical or practical relevance, and results must always be interpreted with caution, taking into account the context and clinical relevance.

Over the years, medicine has evolved from a craftsmanship to an industry, from the world of great masters or great medical schools (*grandes écoles médicales)* to that of recommendations from learned societies or legislators, based on prospective, randomized, and multicenter studies, with their robust methodology, meta-analyses, as well as systematic reviews, with medicine having definitely entered the world of “noble” sciences, just like physics, chemistry, mathematics, and so on [6]. The doctor has been treated the same as the scientist. Yet, the individual must be considered, especially the patient, and what about the existing inequalities in terms of healthcare access and medical progress [7,8]?

Medical journals have similarly “brought to light” molecular biology, as well as advances in technology, pharmacology, and pharmacopoeia, with clinical issues at times being inappropriately “left out in the cold” [9].

With the development of data science [10] and digital technology [11], we are now witnessing a certain return to the past (or rather to the future), to the individual, and to a more personalized, patient-centered approach to healthcare with “P-Medicine”, for Personalized, Preventive, Participatory, Predictive, and Proven medicine, being central (Figure 1) [12]. Thus, in our opinion, medical journals must promote this type of approach and encourage the publication of work that meets the objectives of “P-Medicine”. Using artificial intelligence algorithms, doctors are now in a position to develop more personalized treatment plans based on the patient’s individual characteristics, genetic data, and prior treatment responses.

For the future, it is important to create for each individual patient his or her digital twin [13], coupled with the availability of a bio-bank integrating genomic, proteomic, and other data, making it possible to envisage advanced or improved humans, in addition to the development of trans-humanism [14,15]. 

Given this context, medical journals must likewise integrate sustainable development into their fields to “highlight” the “OneHealth” concept (Figure 1) [16]. This concept promotes close interconnection among human, animal, and environmental health. It promotes a holistic approach to health that recognizes that human health is intrinsically linked to that of animals and that of the ecosystem in which they live. Here too, the board of the *J. Clin. Med*. is particularly attentive to compliance with these recommendations.

Finally, all medical journals need to find the best communication tools and vectors for reaching their readership, disseminating and advancing knowledge, and determining their business model (Figure 1). This is particularly important in terms of their longevity.

## 3. Conclusions

Overall, nowadays, medical journals are a vital channel for the transmission, validation, and discussion of medical knowledge. These journals constitute an essential pillar of the medical community, contributing to the continuous improvement of both healthcare and scientific progress within the medical field. Medical journals are both engines and vectors of medical innovation.

Medical journals still play a crucial role in building a solid knowledge base, promoting new ideas, and creating an environment conducive to continuous innovation in medicine.

Medical journals must, however, adapt to societal evolution and current medical issues; they need to evolve and incorporate the expectations and aspirations of healthcare professionals, patients, and wider society alike, while integrating the “new kings” of the medical world, prominent individuals in medical articles and congresses, including data, artificial intelligence, sustainable development, transhumanism, and last, but not least, the OneHealth concept.

Links of interest: none directly relevant to the content of this manuscript; Professor E Andrès is Editor-in-Chief of several medical journals, including the *J. Clin. Med.*
